# Cell-derived biomimetic nanocarriers for targeted cancer therapy: cell membranes and extracellular vesicles

**DOI:** 10.1080/10717544.2021.1938757

**Published:** 2021-06-18

**Authors:** Aixue Li, Yunan Zhao, Yixiu Li, Liangdi Jiang, Yongwei Gu, Jiyong Liu

**Affiliations:** a College of Pharmacy, Shandong University of Traditional Chinese Medicine, Jinan, Shandong, China; b Department of Pharmacy, Fudan University Shanghai Cancer Center, Shanghai, China; c Department of Oncology, Shanghai Medical College, Fudan University, Shanghai, China; d Department of Pharmacy, Shanghai Integrated Traditional Chinese and Western Medicine Hospital, Affiliated to Shanghai University of Traditional Chinese Medicine, Shanghai, China

**Keywords:** Biomimetic nanocarriers, targeting, cell membranes, extracellular vesicles, exosomes, cancer therapy

## Abstract

Nanotechnology provides synthetic carriers for cancer drug delivery that protect cargos from degradation, control drug release and increase local accumulation at tumors. However, these non-natural vehicles display poor tumor targeting and potential toxicity and are eliminated by the immune system. Recently, biomimetic nanocarriers have been widely developed based on the concept of ‘mimicking nature.’ Among them, cell-derived biomimetic vehicles have become the focus of bionics research because of their multiple natural functions, such as low immunogenicity, long circulation time and targeting ability. Cell membrane-coated carriers and extracellular vesicles are two widely used cell-based biomimetic materials. Here, this review summarizes the latest progress in the application of these two biomimetic carriers in targeted cancer therapy. Their properties and performance are compared, and their future challenges and development prospects are discussed.

## 1. Introduction

Cancer is one of the most invasive diseases and the leading cause of death in humans, and a large amount of effort and money has been devoted to fighting it over the past few decades (Roma-Rodrigues et al., 2019). In the struggle with cancer, early detection is important for successful treatment, and surgery, phototherapy, chemotherapy, radiotherapy (RT) and multimodal combination therapy are the principal methods of cancer treatment used currently. However, surgical resection causes relapse easily after the operation due to the limitation of the tumor distribution. Phototherapy, chemotherapy and RT may have serious side effects due to the lack of specificity (Hu et al., 2016).

In recent years, drug delivery systems (DDSs) based on nanocarriers have been widely developed, and antitumor drug carriers (such as liposomes (Wang et al., 2020), micelles (Guan et al., 2020), polymer nanogels (Maiti et al., 2018), magnetic nanoparticles (NPs) (Avval et al., 2020), and nanocapsules (de Cristo Soares Alves et al., 2020)) are usually produced on the nanoscale to increase their permeability and retention in tumor tissues. However, these carriers are identified as ‘nonself’ and are still quickly cleared by the immune system with a short half-life. They are usually unable to actively sense the disease environment and do not effectively accumulate in the tumor site; thus, their targeting rate is extremely low. In addition, NPs dispersed in vivo adsorb proteins to form a protein corona on their surfaces, which reduce the targeting rate of NPs and mediate the clearance of NPs by the reticuloendothelial system (RES). The polyethylene glycol (PEG) modification can delay the clearance time, but repeated use will induce the production of its specific antibody IgM and accelerate the clearance of NPs (Li et al., 2018). Therefore, traditional nanocarriers still have many defects and are unable to meet the current need for cancer treatment.

Based on the concept of ‘mimicking nature,’ biomimetic nanocarriers have been widely developed due to their advantages in reproducing the functions of natural materials, among which cell-derived biomimetic carriers are currently a hot spot. Due to the different proteins and carbohydrates on different cell membranes, cells perform a variety of specific functions in the body, and thus the nanocarriers formed by cell-derived membranes that encapsulate cargoes may inherit the functions of the source cells, such as immune escape, long circulation, and recognition ability. Therefore, this cell-derived biomimetic carrier is a ‘natural treasure,’ among which the cell membrane and extracellular vesicles (EVs) are widely used.

**Figure 1.**
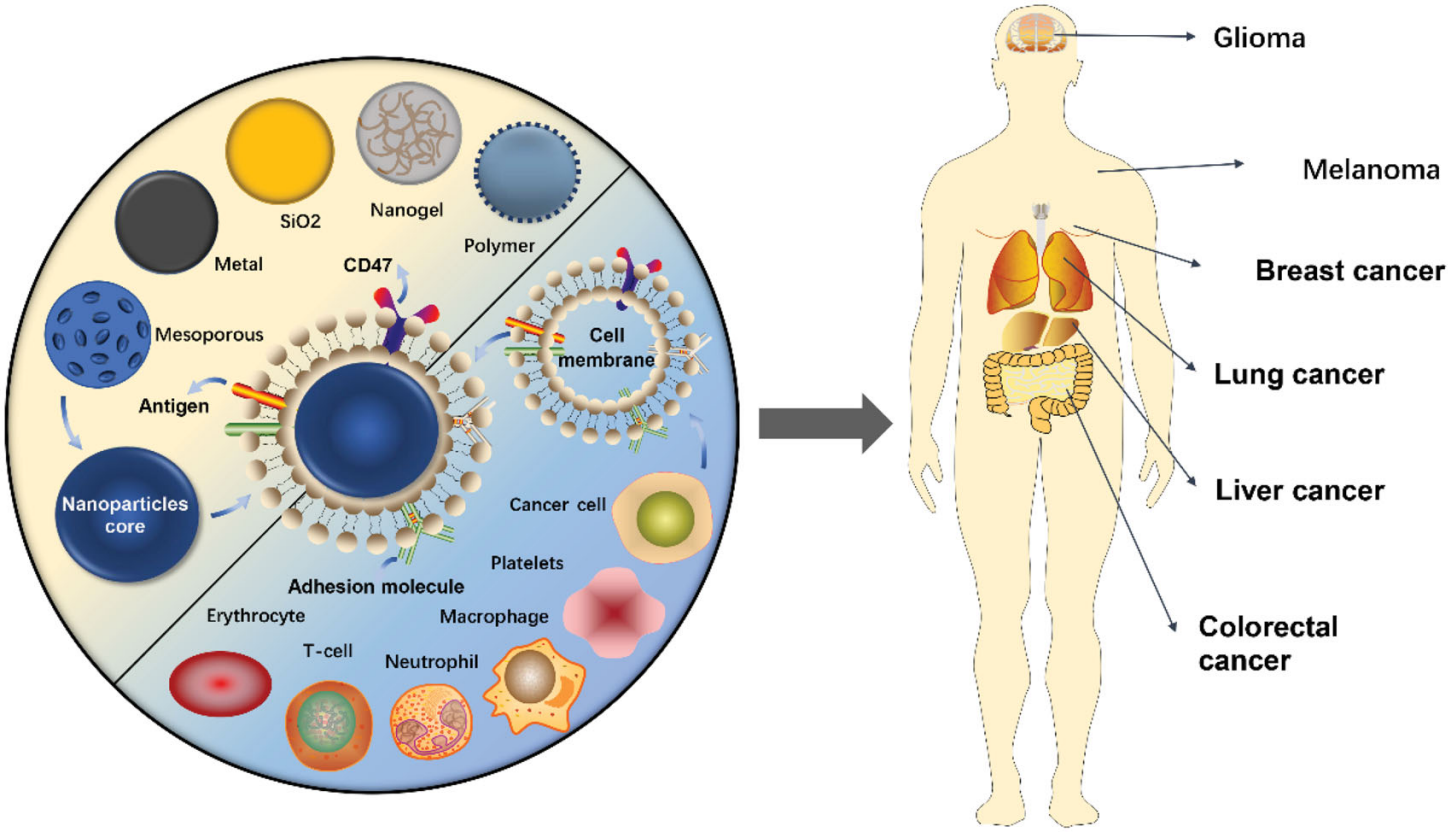
Cell membrane-coated nanoparticles for cancer drug delivery. Cell membranes extracted from different types of cells are used to encapsulate different types of nanoparticles for cancer treatment.

Cell membrane coating technology involves organic/inorganic synthetic NPs wrapped in a layer of natural cell membrane, and the prepared nanocarriers not only function as synthetic NPs but also have the natural complex characteristics of the source cells (Figure 1). Since researchers have successfully prepared red blood cell membrane (RBCM)-coated NPs that prolonged the circulation time in vivo in 2011, cell membrane camouflage technology has developed rapidly (Hu et al., 2011). The function of the cell membrane varies by cell type; for example, red blood cells (RBCs) evade the immune system due to the expression of CD47 on the membrane but have no targeting ability, whereas white cell membranes display the characteristic of tumor homing (Gao et al., 2020; Huang et al., 2018). Therefore, for different application purposes, researchers began to utilize a variety of cell membranes to camouflage NPs, such as the immune cell membrane, platelet membrane (PLTM), and even cancer cell membrane (CCM). The coating of the cell membrane endows NPs with good biocompatibility and adjustable surface properties, suggesting that this biomimetic carrier coated with a cell membrane is extremely promising for cancer therapy (Xu et al., 2020).

**Figure 2.**
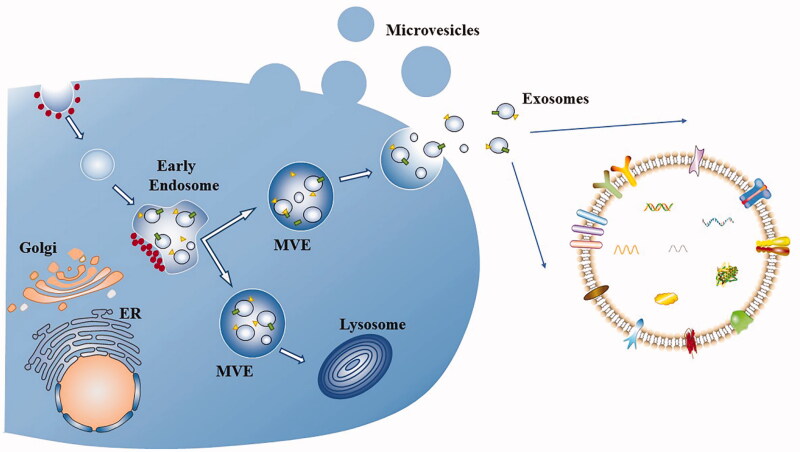
Biogenesis of extracellular vesicles and exosomes. Microvesicles bud from the plasma membrane. Exosomes are small vesicles that form early endosomes and multivesicular endosomes (MVEs), which are released through the fusion of MVEs with the plasma membrane. Other MVEs enter the lysosome. Dots represent clathrin-coated vesicles or clathrin coats, rectangles and triangles represent transmembrane proteins and membrane-associated proteins, respectively.

**Figure 3.**
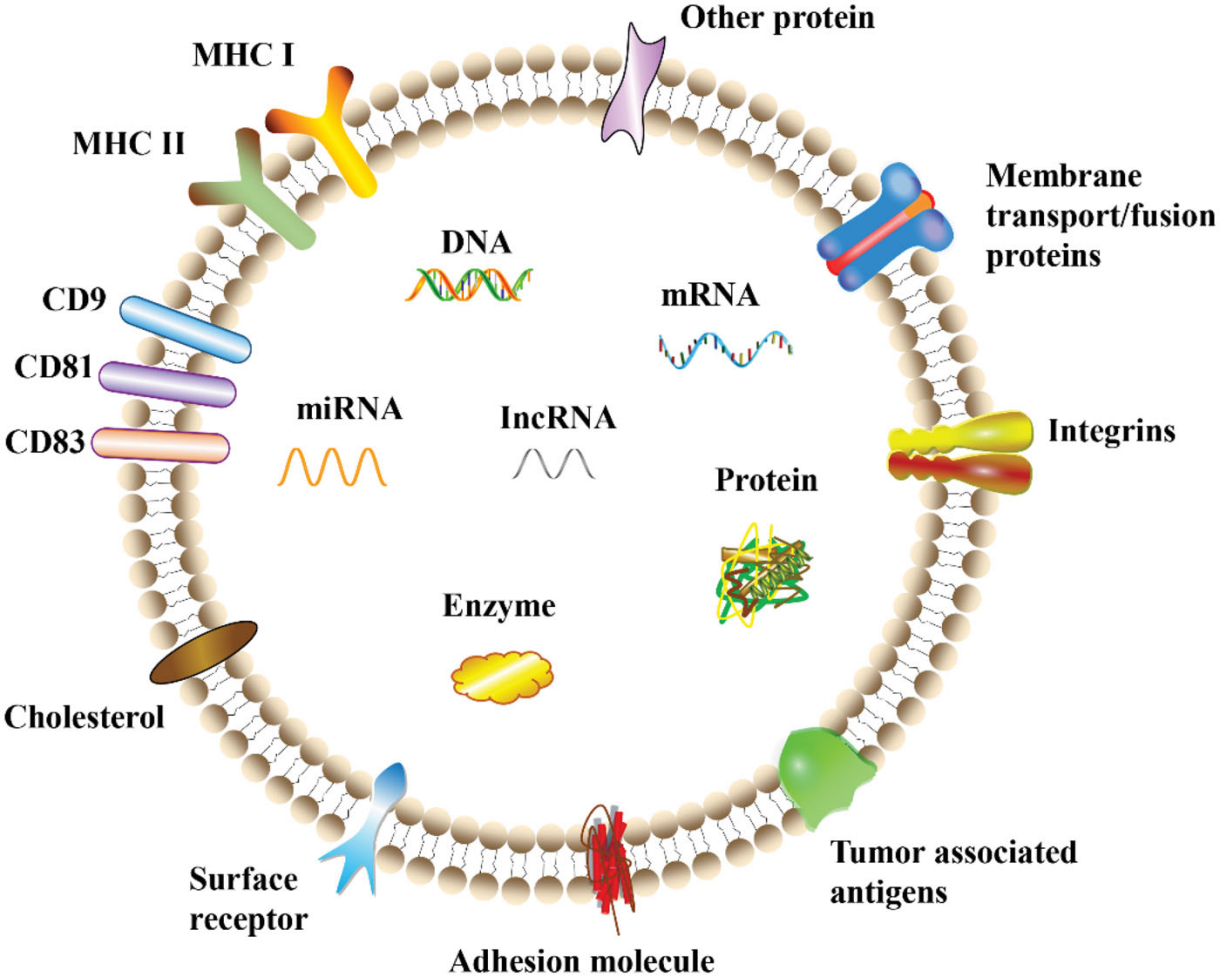
Structure and composition of exosomes. Exosomes are approximately round vesicles secreted by cells that contain various cellular components, including proteins, miRNAs, mRNAs, lncRNAs, enzymes, carbohydrates, and lipids. Various proteins are present on the surface of exosomes and are responsible for different pathophysiological functions.

EVs are phospholipid bilayer vesicles that are secreted by almost all cells, are responsible for intercellular communication and cargo transport, and potentially alter the phenotype of recipient cells. EVs are classified according to their intracellular sources, from small to large, as exosomes (40–120 nm), microvesicles (50 nm–1 μm), and apoptotic bodies (50 nm–5 μm) (Willms et al., 2018). Exosomes and microvesicles originate from endosomes and plasma membranes, respectively, while apoptotic bodies are produced through sprouting and abscission mechanisms and autophagosome formation mechanisms, among which exosomes are the most comprehensively studied vesicles (Figures 2 and 3). However, due to their small size and overlapping dimensions, a precise method is unavailable to distinguish them, and thus these vesicles are collectively referred to as EVs (Zaborowski et al., 2015). EVs have been used as carriers of various biomolecules, such as DNA, RNA and protein. Usually, after the release of EVs wrapped in unique biomolecules, EVs are internalized by their targeted recipient cells and then deliver their contents and transmit genetic information. EVs protect the cargo from degradation during delivery and cross various barriers, such as the blood-brain barrier (BBB), spread to the interior of the body and reach distant tissues (Gargiulo et al., 2019). Importantly, autologous EVs lack immunogenicity and are extremely safe as drug delivery carriers. Building on the advantages described above, EVs have become a ‘new star’ in the field of drug delivery carriers in recent years.

In this review, we summarize the latest applications of cell-derived biomimetic nanocarriers (cell membrane and EVs) as targeted cancer therapy delivery vehicles, compare the differences between these two types of carriers, and discuss their challenges and future development prospects.

## 2. Cell membrane-coated nanoparticles

### 2.1. Preparation of cell membrane-coated nanoparticles

According to the different treatments of diseases, the cell membranes of RBCs, immune cells, platelets (PLTs) and cancer cells can be used to camouflage drugs or NPs as needed. The basic production method is similar and mainly includes the extraction of the cell membrane, the preparation of the nanocore and the assembly of the ‘shell-core’.

#### 2.1.1. Isolation of the cell membrane

Cells perform various complex functions in the body by interacting with the surrounding environment, and most of the responsibilities are performed by functional surface proteins. The properties of proteins on the membrane are easily changed, and thus the extraction and separation of the cell membrane should be carried out carefully. The separation of the cell membrane is the process of separating the membrane and intramembrane mixture, and the extraction of all cell membranes requires cell fragmentation in hypotonic solution, but the separation process for enucleated cells is slightly different from that for eukaryotic cells. The former are often mentioned as RBCs and PLTs, which are directly separated from the blood, and after the cells are lysed, the cell membranes are separated from the mixture by centrifugation. The latter, such as cancer cells and white blood cells (WBCs), are first ruptured with a hypotonic solution, and then their contents are thoroughly separated from the membrane by ultrasound and homogenization. Finally, pure cell membranes are extracted by high-speed differential centrifugation (Su et al., 2017; Fang et al., 2018). Importantly, membrane extraction should be performed under suitable conditions to ensure that the proteins on the membrane maintain their original activity, and protease inhibitors are usually used in extraction.

#### 2.1.2. Fusion of membrane vesicles to the core nanoparticles

The membrane is wrapped around the core through various methods, all with the ultimate goals of maintaining the membrane in the right direction and exposing the proteins on the membrane for communication. Shell-core fusion was first performed using a physical extrusion method that extends from the preparation of liposomes. The extrusion force can destroy the membrane structure and cause it to reform around NPs (Hu et al., 2015). Ultrasonic treatment is also a feasible method. The breaking force generated by ultrasonic energy can cause the cell membrane to spontaneously reshape on NPs (Copp et al., 2014). Ultrasonic treatment exerts the same effect as physical extrusion, but it results in less raw material loss and is easier to expand to the production scale, suggesting that the application prospects are brighter. The semistable characteristics of the membrane and the core, as well as the asymmetry of the charge on the surface of the membrane, help the membrane wrap the nanocore. The special right-side-out membrane orientation makes the combination of the two extremely thermodynamically stable.

Usually, the nanocore is destroyed easily by ultrasonic treatment and time and labor are wasted when using physical extrusion, while microfluidic electroporation, a popular method, can remedy these deficiencies. A microfluidic chip reduces the applied voltage of electroporation. Rao et al. developed a microfluidic chip. When flowing through the electroporation area, the electric pulse promoted Fe_3_O_4_ magnetic NPs to enter the RBC vesicle, which effectively combined the two. The biomimetic carrier produced using this method shows excellent application potential in tumor diagnosis and treatment (Rao et al., 2017). In addition, an emerging cell membrane-templated polymerization technology achieves efficient membrane wrapping and controls the size of the formed biomimetic NPs, but related research is limited and requires further development (Zhang et al., 2015).

### 2.2. Cell membrane-coated nanoparticles for targeted cancer therapy

#### 2.2.1. Red blood cell membrane

RBCs are abundant in the blood and are responsible for transporting oxygen. They have variable shapes and no nuclei and are easily separated from the blood, which facilitates the extraction and purification of cell membranes. RBCs also have a long life span (120 days) and can serve as carriers to improve the biosafety of DDSs and prolong their blood circulation time. However, the lack of cell adhesion molecules on RBCMs prevents them from targeting the tumor tissue, leading to hamper cellular internalization.

RBCMs are often modified with ligands (such as folic acid) to expand the applications of this natural carrier, and two main methods of active targeting modification have been developed. One is the direct modification method of binding ligands to the RBCM with active groups through covalent bonds, but the reagent used to induce binding may destroy the function of RBCM (Chai et al., 2017). Another method is the indirect modification, in which positively charged ligands (lipids or proteins) are inserted into the membrane, but the ligands are easily absorbed by negatively charged RBCMs, and thus the targeting ability is difficult to guarantee (Fang et al., 2013). A recent study cleverly avoided the charge problem by using RBCM to achieve targeted drug delivery for gliomas. In this study, a novel biomimetic carrier (T7/NGR-RBCSLNs) with double modification was constructed by modifying the RBCM with the negatively charged T7 peptide and negatively charged polypeptide NGR through lipid insertion. The former targets transferrin receptors on both the BBB and glioma surface, and the latter targets CD13, which is expressed at high levels in tumor cells. Compared with biomimetic NPs modified with only one ligand, the endocytosis of T7/NGR-RBCSLNs by glioma cells was the most significant. T7/NGR-RBCSLNs take advantage of the dual targeting effect of modified RBCMs to cross the BBB and the blood-brain tumor barrier (BBTB) and significantly enhance the anti-glioma effect *in vivo* (Fu et al., 2019).

In addition to ligand modification, the hybridization of RBCMs with other cell membranes also improves the targeting ability. Because the membrane proteins on PLTs bind to biomolecules expressed at high levels in some tumors, Kim et al. prepared a new biomimetic carrier (R/P-cGNS) that used gold nanostars loaded with curcumin (Cur) as the core, and the cloak was a mixture of RBCMs and PLTMs. R/P-cGNS has two membrane functions, because the carrier not only escapes phagocytosis but also effectively targets tumors (Kim et al., 2020). Natural cell membranes are affected by temperature. Combined with photothermal therapy (PTT), R/P-cGNS achieves the controlled release of Cur with increasing temperature to achieve the expected anticancer effect (Ebrahimi et al., 2018).

RBCMs were natural, abundant and safe, and can be used as a favorable antitumor tool after being endowed with target ability (Yu et al., 2019). However, besides that, the quality control of RBCs is also a challenge. It is necessary to ensure that the RBCMs will not be contaminated by pyrogens and viruses, to remove the deformed proteins, and to avoid the potential immune reaction of endogenous antigens (Li et al., 2018). For further clinical studies, the RBCMs should be matched to the patient's blood type and RH compatibility (Han et al., 2018).

#### 2.2.2. White blood cell membrane

WBCs, also known as immune cells, are nucleated, colorless, spherical blood cells that migrate freely inside and outside blood vessels, widely exist in blood, lymph and various tissues, and affect the progression of various diseases. WBC membrane-camouflaged NPs, which endow NPSs with both an immune escape ability and active targeting ability, have been widely used as drug delivery carriers in recent years (Li et al., 2018). Macrophages and neutrophils (NEs) are the most commonly utilized WBCs.

According to the different activation states, macrophages are divided into M1 and M2 macrophages. M1 macrophages exert proinflammatory effects, induce a positive immune response and destroy tumor tissue, while M2 macrophages exert anti-inflammatory effects, downregulate the immune response and promote tumor growth (Shapouri-Moghaddam et al., 2018). The antitumor effect of M1 macrophages is mainly derived from their surface markers, such as major histocompatibility complex II (MHC-II), CD80, and CD86, and thus antitumor carriers based on macrophage membranes have been widely developed (Najafi et al., 2019). However, macrophages are affected by the complex tumor microenvironment (TME), and the antitumor effect must often be enhanced by combining macrophages with other therapies. Hu et al. prepared biomimetic nanocarriers encapsulated by the M1 macrophage membrane [(C/I)BP@B-A(D)&M1m]. Various molecules involved in costimulatory signal transduction and high expression of MHC on the cell membrane allowed (C/I)BP@B-A(D)&M1m to effectively target tumor tissues. Combined with laser irradiation, (C/I)BP@B-A(D)&M1m released drugs efficiently at the target site as needed (Hu et al., 2020). Liu et al. developed a mixed micelle with photosensitizer chlorin e6 (Ce6) and reactive oxygen species (ROX) responsive bilirubin, loaded with modified paclitaxel (PTX) dimer, and coated with macrophage membrane (I-P@NPs@M). I-P@NPs@M effectively combining chemotherapy and photodynamic therapy (PDT) by co-delivering Ce6 and PTX. Macrophage membrane can protect drugs from the capture by mononuclear macrophage system, which makes I-P@NPs@M more to be absorbed and retained by tumor cells (Liu et al., 2019; Liu et al., 2020).

**Figure 4.**
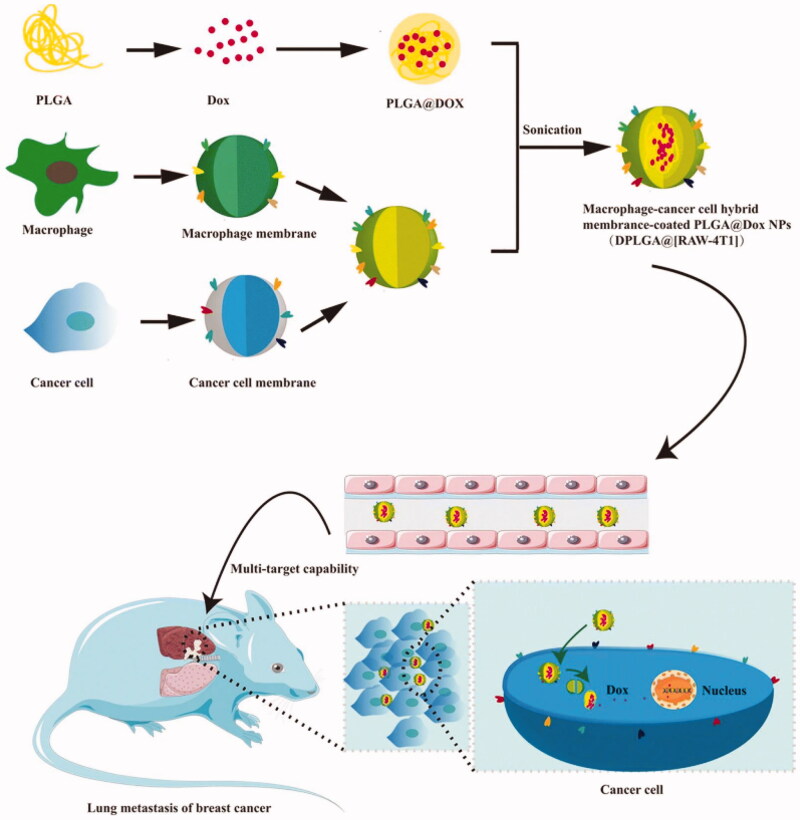
Formation and release of RAW-4T1 hybrid membrane-coated doxorubicin (Dox)-loaded PLGA nanoparticles (DPLGA@[RAW-4T1] NPs). Reproduced with permission from Reference (Gong et al., 2020).

Macrophages regulate various functions in tumor immunity, not only participating in early cancer but also affecting the metastasis of terminal cancer (DeNardo and Ruffell, 2019; Jäppinen et al., 2019). Gong et al. loaded doxorubicin (Dox) into poly(lactic-co-glycolic acid) (PLGA) NPs and coated them with a hybrid coating of macrophage (RAW264.7) membranes and breast cancer cell (4T1) membranes to form new biomimetic nanocarriers (DPLGA@[RAW-4T1] NPs) (Figure 4). The α4β1 integrin on the RAW264.7 membrane is activated by vascular cell adhesion molecule-1 (VCAM-1), which is expressed at high levels on metastatic cancer cells, thereby increasing the ability of DPLGA@[RAW-4T1] NPs to specifically target metastatic cancer tissue. The 4T1 membrane enables DPLGA@[RAW-4T1] NPs to target homologous cancer cells, efficiently track the tumor and kill the tumor tissue (Gong et al., 2020). This biomimetic carrier is the first attempt to combine the macrophage cell membrane with CCM, which assists in the treatment of metastatic breast cancer and prolongs the life of patients, indicating its promising application prospects. However, whether macrophages from different individuals or races will produce individual immune rejection remains to be explored.

NEs, the most abundant immune cells, are the first to respond to infection or tumors and are closely related to tumor progression; thus, they are potentially useful as excellent carriers of antitumor drugs (Han et al., 2018; Zhang et al., 2020). Glioblastoma grows rapidly and has a high fatality rate, increasing the difficulty of complete surgical resection, and the BBB and BBTB also prevent common chemotherapy drugs from easily passing through and reaching the tumor site, resulting in a high tumor recurrence rate. Typically, postoperative inflammation occurs at the glioma site, and inflammatory cytokines [interleukin-8 (IL-8) and tumor necrosis factor α (TNF-α)] at the inflammation site activate NEs and induce their migration to the inflammatory site. Zhao et al. prepared a novel NE-based biomimetic carrier (PTX-CL/NEs) by wrapping neutrophil membranes (NEMs) around liposomes loaded with PTX. PTX-CL/NEs effectively target postoperative tumor sites where inflammatory signals are amplified, release drugs effectively, and slow tumor recurrence and growth (Xue et al., 2017). In another study, NEMs were wrapped on Celastrol-loaded PEG-PLGA nanoparticles. NEMs allow the nanoparticles to be recruited by chemokines, cross the blood-pancreas barrier and metastasize to the tumor site, thereby effectively exerting an antitumor effect (Cao et al., 2019). Although NEs are rich in content and fast in recruitment, they have a short lifespan, so they are often used in acute treatment environments (Combes et al., 2020).

In summary, immune cell membrane-based carriers with good tumor recognition ability and tumor penetration play a major role in regulating tumor occurrence and metastasis. Combined therapy with other methods is more effective. However, WBCs are highly heterogeneous. If allogeneic blood is used as a membrane source, blood type compatibility tests and screening for infectious diseases are required (Wu et al., 2019). In addition, the complexity of the TME makes it difficult to accurately grasp the mechanism of immune cell recruitment and polarization, which is still a challenge for effective drug delivery.

#### 2.2.3. Platelet membrane

PLTs are disc-shaped and changeable, with a diameter of approximately 1-4 μm, and express membrane proteins such as p-selectin and CD47, which identify injured blood vessels and circulating tumor cells (CTCs). Because PLTs aggregate at the tumor site and their cell membranes are easy to extract and purify, the number of PLT-based drug delivery schemes has increased rapidly in recent years (Chen et al., 2018). Wang et al. encapsulated black phosphorus quantum dots with drug-carrying PLTM to form a new carrier (PLT@BPQDs-HED). The fluorescence signal of PLT@BPQDs-HED was stronger at the tumor site, and the retention rate was significantly higher after 48 hours than the control group. Because p-selectin on PLTs selectively binds to the overexpressed CD44 receptor on the tumor surface, PLT@BPQDs-HED has a higher efficiency of tumor drug delivery and enables the drug remain in the target site (Shang et al., 2019).

PTT is an invasive therapy used in combination therapy (Chen et al., 2019; Liu et al., 2019), but nanocarriers loaded with photothermal materials are unable escape the recognition of immune cells and are quickly cleared, resulting in low therapeutic efficiency. Wu et al. camouflaged NPs containing polypyrrole (PPy) and the anticancer drug Dox with the PLTM (PLT-PPy-Dox). The membrane protein enables PLT-PPy-Dox to evade the attack of immune cells and precisely target the tumor, laser irradiation causes PPy to produce hyperthermia and ablate the tumor cells, and Dox is also released from the NPs to effectively destroy the tumor. In addition, after three consecutive PTT cycles, the temperature of the tumor tissue increases, and then the tumor cells are burned; Due to PLTM recognize and accumulate in the injured site, PLT-PPy-Dox is more gathered in the tumor site, which promotes the efficiency of PTT (Wu et al., 2020). Therefore, the strategy of using biomimetic materials and combination therapy in the treatment of tumors has significant effects and promising prospects.

Cancer immunotherapy is a new method to stimulate the autoimmune response to destroy tumors, including monoclonal antibodies, cancer vaccines, and immune checkpoint inhibitors, among which the application of immune checkpoint blockade therapy in cancer treatment has been gradually developed in recent years (Friedman et al., 2020; Veldman et al., 2020). However, the TME is extremely complex, and tumor cells protect themselves from attack in various ways, limiting the effectiveness of immunotherapy (Phuengkham et al., 2019). Jiang et al. combined mild immunogenic ferroptosis with programmed cell death 1 (PD-1) immune checkpoint blockade therapy to treat cancer, and his team prepared Fe_3_O_4_ magnetic NPs loaded with sulfasalazine (SAS) and coated with PLTM (Fe_3_O_4_-SAS@PLT). The expression of p-selectin on PLTM enables Fe_3_O_4_-SAS@PLT to target tumors in mice and accumulate in the tumor site, thereby inducing ferroptosis and triggering the immune response. Twenty-four hours after injection, a high level of signal was still detected in the tumor site, indicating that the protection of PLTM enabled Fe_3_O_4_-SAS@PLT to escape the ‘pursuit’ of immune cells and facilitated a long circulation time. In addition, the immune response induced by Fe_3_O_4_-SAS@PLT enhanced the efficacy of PD-1 inhibitors, and almost no tumor metastasis occurred in metastatic mice (Jiang et al., 2020).

PLTs are closely related to tumor cells, and carriers that wrap the PLTM are not only treated as ‘self’ by the immune system to avoid clearance but also target tumors through their membrane surface proteins. PLTM-based DDSs may be combined with phototherapy, immunotherapy and other methods to effectively target CTCs to control the development of tumors and have broad application prospects in the treatment of tumors. However, the relationship between PLTs and tumor cells has not been fully elucidated, especially the role of PLTs on cancer cells beyond the function of blood metastasis; thus, sophisticated models that accurately mimic human disease are needed to unravel the variable interactions of PLTs in different cancers (Hyslop and Josefsson, 2017).

#### 2.2.4. Cancer cell membrane

Cancer cells can replicate indefinitely, are easy to culture in vitro, and a large amount of membranes can be isolated from these cells. The CCM is rich in functional proteins, and its molecular repertoire is divided into three categories according to its use: (1) membrane proteins that mediate homotypic binding, such as selectin, tissue factor-antigen, and integrins (2) markers that promote immune escape, such as CD47; and (3) unique tumor antigens that stimulate the immune response of the body (Fang et al., 2018; He et al., 2020; Janiszewska et al., 2020). Given these advantages, the CCM has attracted the attention of researchers, and the use of CCM-modified NPs for drug delivery in tumor therapy is promising.

Although many treatments have been developed for cancer, chemotherapy is still the most common treatment. However, the efficiency of chemotherapy is often reduced by multidrug resistance (MDR), which is a difficulty that researchers have been attempting to overcome (Dei et al., 2019; Negi et al., 2019). CCM encapsulates calcium channel antagonists to overcome MDR by regulating intracellular channels in tumor cells. A recent study developed multidrug-resistant cervical cancer cell (HeLa/Dox) membrane-decorated silica NPs for the codelivery of siRNA and Dox (CCM/CS/R-D). The siRNA replaces the commonly used Cav antagonist, reduces the Ca^2+^ level, and increases the number of cells in the DNA synthesis stage, thus increasing drug retention. Ability of multidrug resistant CCM to bind to homotypic cell membranes and the integrin-associated protein CD47 on CCM endows CCM/CS/R-D with an excellent targeting ability and *in vivo* escape ability, which are conducive to the efficient arrival and function of DOX and siRNA in tumors (Zhao et al., 2020).

**Figure 5.**
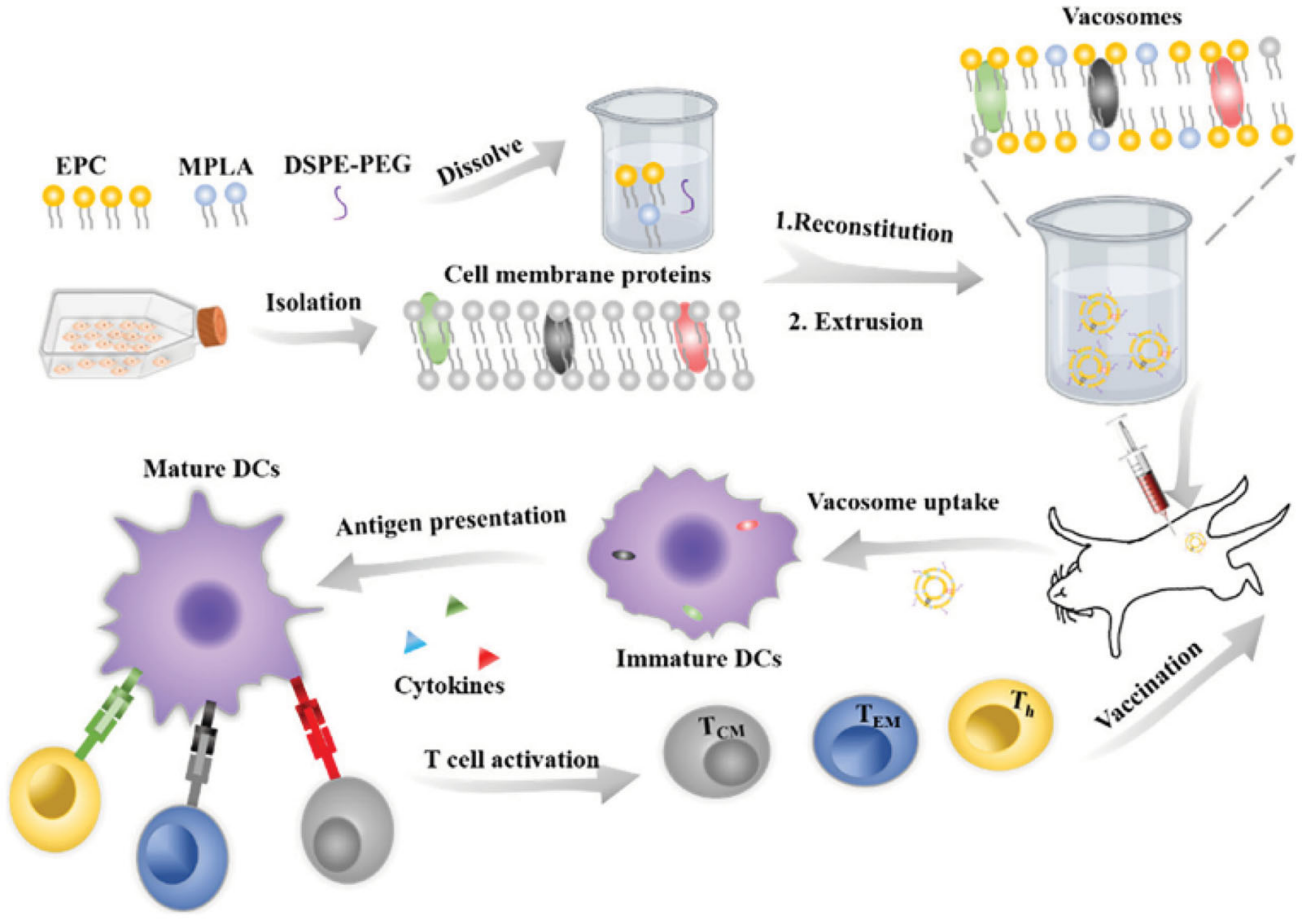
Process used to fabricate the vacosome and the immune response induced by the vacosome *in vivo*. Reproduced with permission from reference (Cheng et al., 2020).

Cancer vaccines usually resist cancer cells by stimulating the human immune system, which is an effective method of cancer immunotherapy (Hu et al., 2018; Fusciello et al., 2019). CCM-coated nanoscale drugs have significant potential as cancer vaccines due to the unique tumor antigens of source cells (Zhu et al., 2017). A cancer vaccine (Vacosome) was prepared by mixing the 4T1 cell membrane, Toll-like receptor agonist monophosphoryl lipid A (MPLA) and common lipids in a certain proportion (Figure 5). The presence of tumor-specific antigens on the CCM enables MPLA to activate the corresponding receptors and increase the activity of antigen-presenting cells (APCs), thereby activating CD8^+^ T lymphocytes (TLs) to destroy tumors. The CCM in Vacosome is obtained from the tumor cells of patients after surgery, and thus the development of Vacosome is more personalized and more widely used in cancer immunotherapy (Cheng et al., 2020). At present, nanovaccines are still in the early stage, some problems remain to be solved (for example, the antigens on the membrane may be degraded in the complex physiological environment), and the effectiveness of their use must be optimized.

This unique yolk-shell structure based on the CCM has promoted progress in various cancer treatment methods with high application flexibility, and its unique membrane components endow the carrier with various capabilities, improve the transport path of the carrier in the body, and improve the efficiency of tumor treatment. In addition, the use of primary tumor cell membranes to develop more personalized and novel treatments is one of the important directions for the future development of CCM systems. The challenge is the reproducible synthesis of CCMs under good medical practice conditions and a deep understanding of their underlying homologous targeting mechanisms. Other important characteristics, such as purity, safety, and integrity, need further clarification (Jin and Bhujwalla, 2019).

#### 2.2.5. Other cell membranes

The most commonly used types of cell membranes have been discussed above. With the development of biomimetic science, an increasing number of cell membranes have been employed for drug delivery. Researchers have also used activated fibroblast (AF) membranes to camouflage NPs in cancer treatment. Because tumor-associated AFs secrete growth factors and cytokines, interact with tumor cells and promote the progression of cancer, the modification of the AF cell membrane on the surface of NPs enables NPs to target tumor-associated AFs and improve the efficiency of various cancer treatment methods (Li et al., 2018). In addition, mesenchymal stem cells (MSCs) with strong self-renewal capacity and myeloid-derived suppressor cells (MDSCs) have also been used to carry nanodrugs to target the TME (Liu et al., 2020; Tesi, 2019).In addition, scientists are increasingly focusing on bacterial cell membranes. Ye et al. prepared a new nanomedicine using PC7A and CPG oligodeoxynucleotides as the core and a maleimide-modified *Mycobacterium smegmatis* (MS) membrane as the shell. MS are nonpathogenic bacteria with strong immunogenicity, and after RT treatment, the MS membrane captures neoantigens produced by tumor cells, promotes the uptake of these antigens by dendritic cells (DCs), and stimulates the antitumor response of TLs (Patel et al., 2019).

Research on membrane-based DDSs is developing rapidly, and researchers are aggressively exploiting the function of cell membranes (Table 1). In recent years, hybrid membrane-camouflaged nanocarriers have also attracted much attention, and the fusion of different membranes enables carriers to inherit the specific functions and key proteins of the source cells, thus having greater advantages in terms of effectiveness and safety (Jiang et al., 2019). As mentioned above, RBCM can prolong the circulation time of the carrier *in vivo* but lacks targeting ability. Sun et al. have constructed a RBCs-cancer cells hybrid membrane coated Dox gold nanocage for the combined PTT/RT/chemotherapy of breast cancer (Sun et al., 2020). The carrier encapsulated with hybrid membrane inherits the excellent homologous tumor-homing and the immune escape ability of RBCs, and can gather efficiently in the target sites. Bu et al. coated the hybrid membrane of PLTs and tumor stem cells on magnetic iron oxide NPs for the treatment of head and neck squamous cell carcinoma (Bu et al., 2019). PLTM evades the recognition of the immune system through the signal expression of surface markers, while the surface adhesion molecules of tumor stem cells enable the carrier to target tumor cells. In TME, this carrier enhances the tumor magnetic resonance imaging characteristics of NPs and shows a good photothermal effect, which could be an excellent delivery tool for tumor therapy. Therefore, hybrid membrane-wrapped NPs can make up the defects of single-cell membrane and give full play to its advantages in a ‘natural modification’ way, so that the NPs can be endowed with at least two biological functions by the cell membrane, of which targeting is crucial.

**Table 1.** Cell membrane coated nanocarriers as carriers for targeted cancer therapy.

Source cell	Nanoparticles	Cargo	Cancer type	Outcome	Ref
RBCM	Prussian blue nanoparticles	Folic acid and compound(J5)	Cervical cancer	Significantly enhancing the synergistic antitumor effect of phototherapy/chemotherapy	(Daniyal et al., 2020)
RBCM	Upconversion nanoparticles	DSPE-PEG-FA	Breast cancer	Successfully realizing the tumor PET imaging	(Li et al., 2020)
RBCM	Bovine serum albumin nanoparticles	Indocyanine green and gambogic acid	Breast cancer	Significantly improving the antitumor effect of synergistic chemotherapy-photothermal therapy	(Wang et al., 2020)
RBCM	Prussian blue nanoparticles	Hyaluronic acid and gamabufotalin	Breast cancer	Accurately, efficiently and safely treat breast cancer	(Liu et al., 2019)
WBC membrane	Gallium nano-swimmer	Dox	Cervical cancer	Enhanced photothermal and chemical cancer therapy	(Wang et al., 2020)
WBC membrane	Lipid nanovector	Dox and siRNA	Esophageal cancer	Realized targeted therapy of esophageal cancer	(Jun et al., 2020)
WBC membrane	Fe_3_O_4_ magnetic nanoclusters	DSPE-modified SYL3C aptamer	Cancer	Realized the rapid and specific detection of CTCs	(Zhang et al., 2019)
WBC membrane	Bimetallic nanoparticles	Epithelial cell adhesion molecule antibody	Epithelial cancer	Realized to capture and analyze CTCs	(Chang et al., 2020)
Macrophage plasma membrane	Albumin nanoparticles	PTX	Melanoma	Accumulating more at the tumor and exerting stronger antitumor effect	(Cao et al., 2020)
Macrophage membrane	Biomimetic superparticle	Dox hydrochloride and quaternary quantum dots	Lung cancer	Specifically targeting metastatic nodules in the lung	(Liang et al., 2020)
PLTM	Porous nanoparticles	Bufalin	Liver cancer	Inhibiting tumor growth	(Wang et al., 2019)
PLTM	Liposome	Dox	Cancer	Improving antitumor effect	(Liu et al., 2019)
PLTM	Nanostructured lipid nanoparticles	PTX	Ovarian cancer	Targeting and treating tumors effectively	(Bang et al., 2019)
Tumor cell membrane	Aluminum phosphate nanoparticles	CpG	Melanoma	Suppressing tumor progression	(Gan et al., 2020)
Tumor cell membrane	Mesoporous silica nanoparticle	Dox and mefuparib hydrochloride	Breast cancer	Enhanced antitumor activity	(Nie et al., 2020)
Tumor cell membrane	Zeolitic-imidazolate framework hybrid nanoparticle	Cisplatin and oleanolic acid	Bladder cancer	Promoting cell apoptosis and reversing MDR in tumor cells	(Chen et al., 2020)
Tumor cell membrane	PLGA nanoparticles	PTX and siRNA	Cervical cancer	Precisely treating of cervical cancer through chemo-gene combined therapy	(Xu et al., 2020)

Currently, PLTs-RBCs hybrid membranes (Liu et al., 2018), WBCs-cancer cells hybrid membranes (He et al., 2018), macrophage-cancer cells hybrid membranes (Gong et al., 2020), and bacterial outer membrane vesicles (OMVs)-cancer cells hybrid membranes (Chen et al., 2020) have been successfully developed for cancer therapy. However, current studies have focused on the coating of single-cell membranes, and there are still some challenges before functionalizing using multiple cell membranes. This novel hybrid membrane coating strategy opens up a new way to overcome the limitations of the current tumor treatment.

## 3. Extracellular vesicles

### 3.1. Extracellular vesicle isolation and drug loading

**Figure 6.**
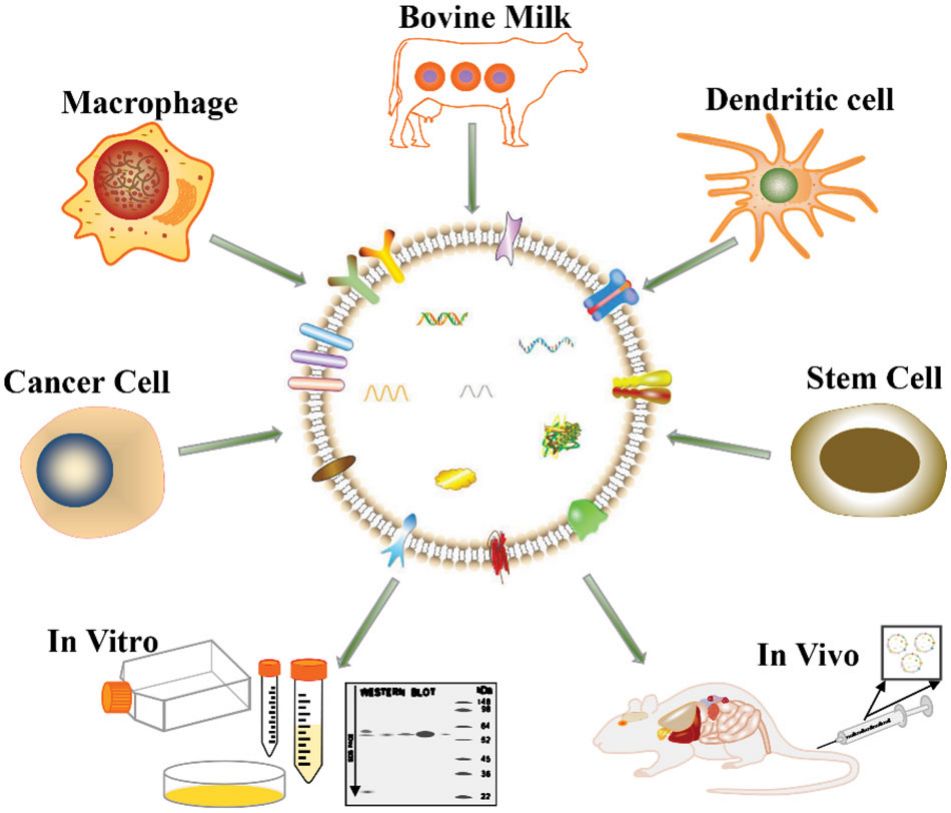
EVs secreted by different cells for targeted cancer therapy.

When EVs are employed for research, appropriate separation methods are often designed according to the research purpose. When EVs are used for diagnosis, a sufficient quantity is more important than purity, and thus a separation method that produces a high yield must be chosen. However, when EVs are used for drug delivery, their structural integrity is extremely important because substances such as proteins on EVs may have roles in targeting. In addition, many types of cells are commonly used to extract EVs (Figure 6), and therefore the separation method should also consider the characteristics of the sample, such as the viscosity of the sample and the concentration of EVs (Abramowicz et al., 2016). Overall, the separation method expected by researchers is simple and less expensive, and EVs are quickly extracted from larger samples. Most traditional separation methods are based on the size and buoyant density of EVs, among which the most commonly used method is ultracentrifugation. Ultracentrifugation has been used to separate EVs from a large number of samples with the consumption of very few reagents and good reproducibility, but the purity of EVs is not high. Density gradient ultracentrifugation improves the efficiency of particle separation and the purity of EVs, but it has the disadvantages of requiring expensive equipment, a long time and a large amount of labor; thus, its clinical application is limited (Tauro et al., 2012). Ultrafiltration is also a commonly used method that saves time and money, but the number of EVs isolated is limited, and the purity is low (Alvarez et al., 2012). However, the combination of ultrafiltration and ultracentrifugation effectively divides different subgroups of EVs, which has promising application prospects (Xu et al., 2017).

The isolated EVs are composed of lipid bilayers that can be used as drug carriers to wrap the cargo and protect it from degradation in vivo. However, the introduction of cargo may destroy the contents and membrane structure of EVs, and thus effective strategies for loading therapeutic cargo into EVs are a challenge. Currently, two main ways for EVs to carry cargo have been developed. One is to combine the source cells of EVs with therapeutic drugs using an endogenous loading mechanism ensuring that the vesicles secreted by cells contain target drugs, of which the most common is the direct transfection of a therapeutic small RNA into cells, and then cells secrete the desired type of EVs (Kosaka et al., 2010). A recent study modified α-fetoprotein (AFP) on DCs, which secrete exosomes carrying AFP, for use in the immunotherapy of hepatocellular carcinoma (HCC) (Lu et al., 2017). The other is the direct combination of curative drugs and EVs, also known as exogenous loading. Hydrophobic drugs, such as Cur and PTX, directly combine with EVs through a coincubation, but hydrophilic compounds, such as RNA, are affected by lipid bilayers, preventing them from being encapsulated in EVs. At present, hydrophilic compounds are loaded into EVs by electroporation and coupled with membrane stabilizers to reduce the effect of the loading process on the structural integrity of EVs (Hood et al., 2014). In summary, the loading method should be selected according to the cargo and specific application purpose, and the cargo loading capacity of different EVs requires further study.

### 3.2. EV-based drug delivery systems

#### 3.2.1. Dendritic cells

DCs are a ‘scout’ among immune cells that accumulate around cancer cells based on the attraction provided by immune signals (such as proinflammatory cytokines and pathogen-associated molecular patterns). These immune signals trigger MCH-1 and MCH-2 on DCs to interact with costimulatory molecules and transport tumor-associated antigens (TAAs) to lymph nodes, where they transmit information to naive TLs, which differentiate into mature TLs and attack cancer cells (Chen and Mellman, 2013). Briefly, the ultimate goal of DCs is to activate TLs to fight tumors, and therefore a promising approach is to design tumor immunotherapy based on the characteristics of DCs. However, the problems that limit the application of DCs, such as high price, short efficacy period and complicated preparation method, still remain to be solved (Palmer et al., 2009). DC-derived exosomes (DEXs) contain the membrane components (for example, DC-originating molecules) of DCs and possess the targeting and immune stimulation capabilities of their source cells. The composition of their membrane is easy to control and stable, and these exosomes can be frozen for half a year (Zhang et al., 2014).

The feasibility of dendritic cell-derived EVs (DC-EVs) for cancer treatment has been proven, but antitumor experiments showed that DC-EVs do not eradicate tumors, and the insufficient induction of the immune response leads to the ability of tumors to overcome immune attack, which is the main reason for the limited therapeutic effect (Viaud et al., 2010). Therefore, DC-EVs with higher immune activity must be developed to combat tumors. In a recent study, ovalbumin (OVA) was added to DC cultures as an antigen, followed by interferon-γ (IFN-γ) and lipopolysaccharide stimulation to activate DCs, and then activated exosomes (DC_OVA_-sEVs) containing ovalbumin were collected. DC_OVA_-sEVs activated by IFN-γ and lipopolysaccharide polarize M2 macrophages to M1 macrophages that are involved in a positive immune response. Because DC_OVA_-sEVs contain antigens, MHC I, MHC II and other molecules, activated DC_OVA_-sEVs interact with TLs, macrophages and DCs to improve antitumor immunity in vivo. DC_OVA_-sEVs increase antigen levels through APC-dependent mechanisms and APC-independent mechanisms, but the former is the major pathway, indicating that the delivery of APCs is the key to obtaining immunity. In addition, DC_OVA_-sEVs do not induce negative phenomena, such as promoting angiogenesis, and the dose used does not cause systemic toxicity, suggesting that they have bright development prospects as a type of DC-EV with high immune activity (Matsumoto et al., 2020).

In addition to delivering proteins as tumor vaccines, DC-EVs can also be directly loaded with drugs for antitumor therapy. In a recent study, DEXs with specific membrane proteins led their loaded fluorouracil (Fu) to directly target cancer cells, fuze with the cell membrane and increase drug internalization, which is expected to replace long-term intravenous administration of Fu and reduce side effects (Xu et al., 2020). However, few studies have examined the direct loading of therapeutic drugs in DC-EVs, and the therapeutic effect of the loaded drug still must be judged based on the results of practical experiments.

#### 3.2.2. Stem cells

**Figure 7.**
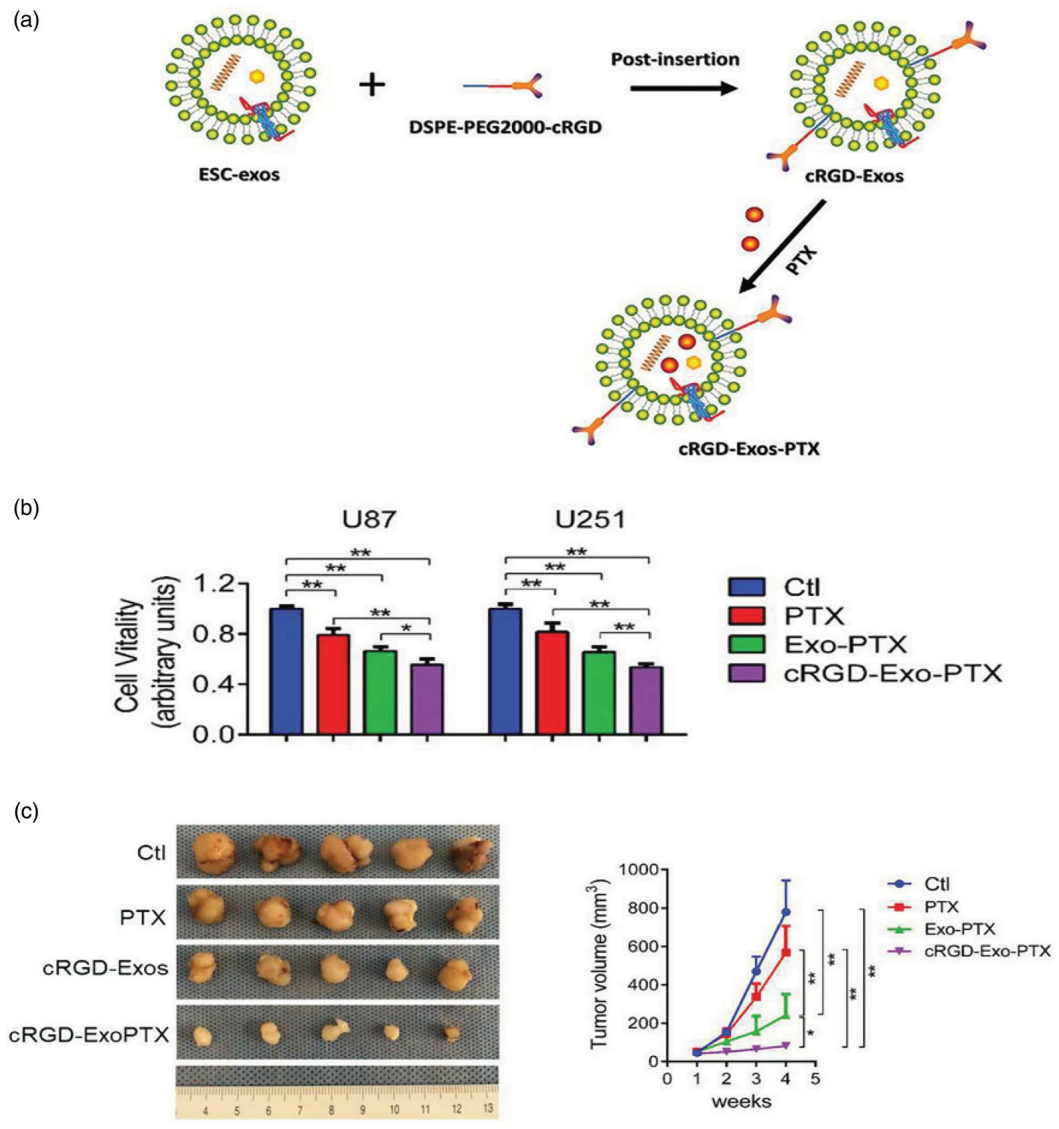
Targeting capability of cRGD-Exo-PTX *in vitro* and *in vivo*. (a) Schematic diagram of the synthesis of cRGD-Exo-PTX. (b) Cell viability of PBS (CTL group), PTX, Exo-PTX, and cRGD-Exo-PTX groups during targeted therapy was determined using the CCK-8 assay. U87 and U251 are two human GBM cell lines. (c) Schematic diagram of subcutaneous U87 GBM in different groups and tumor volumes measured at specified time points during in vitro targeted therapy. Reproduced with permission from reference (Zhu et al., 2019).

One of the important reasons why the problem of GBM is difficult to overcome is that the BBB prevents drugs from reaching the tumor, and the efficiency of various drug carriers at penetrating the BBB is very low. Naturally, exosomes, natural endogenous carriers, are widely used as ideal carriers for treatment due to their nontoxicity, protective drug-carrying capacity and strong penetration of biological barriers. A study took advantage of the unlimited proliferation of embryonic stem cells (ESCs) to mass produce ESC-derived exosomes (ESC-exos) for the treatment of GBM. ESC-exos contain ESC-specific reprogramming factors with antitumor function that reprogram malignant tumors to a benign phenotype (Costa et al., 2009; Díez-Torre et al., 2009; Zhou et al., 2017). A peptide (C (RGKyK)) was modified on the surface of ESC-exos to target the αvβ_3_ integrin receptor that is overexpressed on tumor cells to improve the targeting of these carriers, and then PTX was loaded (Figure 7(a)). The new carrier (cRGD-Exo-PTX) transports drugs to target tumors through the BBB and then releases drugs. Compared with the control group, the cRGD-Exo-PTX group decreased the vitality of GBM more effectively and inhibited the growth of GBM (Figure 7(b,c)). The use of ESC-exos as a drug delivery carrier is a promising treatment for GBM that may play an unexpected role in the treatment of other cancers (Zhu et al., 2019).

MSCs are present in numerous tissues, such as fat and bone marrow, and MSCs from different sites also have distinct gene expression patterns and differentiation potential. Many studies have shown that MSCs are involved in tumor growth (Xie et al., 2019), angiogenesis (Zhu et al., 2012), metastasis (de Araujo Farias et al., 2018), drug resistance (Kabashima-Niibe et al., 2013) and other processes; therefore, MSCs have become a hot topic in cancer treatment. Bone marrow mesenchymal stem cells (BM-MSCs) have unique properties, such as immune regulation and strong self-renewal ability, and BM-MSC-derived exosomes (BM-MSCs-EXOs) have also been proved to have good therapeutic effects. As a messenger, BM-MSCs-EXOs transport various cargoes to receptor cells, including therapeutic drugs and a variety of biological macromolecules, such as RNA (Kabashima-Niibe et al., 2013; Błogowski et al., 2016; Zhou et al., 2020). Recently, antimiR-142-3P was modified with locked nucleic acid (LNA-antimiR-142-3p) and then loaded into BM-MSC-EXOs in an attempt to inhibit the growth of breast cancer stem cells. Due to the homologous targeting of BM-MSCs, the modified BM-MSC-EXOs delivered LNA-antimiR-142-3p to tumor stem cells and suppressed miR-142-3p expression, subsequently reducing the expression of miR-150 and ultimately significantly reducing the proliferation and tumor-initiating capability of tumor stem cells (Naseri et al., 2020). In addition, BM-MSCs-EXOs have low immunogenicity and can modulate the TME that affects tumor growth and development. Ying et al. transfected miR-193a into BM-MSCs-EXOs to target focal adhesion kinase in colon cancer, which downregulated the expression of focal adhesion kinase and inhibited the invasion, migration and proliferation of colon cancer cells (François et al., 2019; Ying et al., 2020). In another study, BM-MSCs-EXOs carrying miR-193A promoted apoptosis in drug-resistant lung cancer cells by downregulating LRRC1 (Wu et al., 2020). However, few studies have examined the transport RNA of BM-MSCs-EXOs to tumor stem cells, which creates new and feasible carriers for tumor drug delivery and has the value of further exploration.

In MSCs, the clinical application of adipose-derived mesenchymal stem cells (ADSCs) is widely used in the clinic, and it is one of the hotspots of tumor treatment (Miana and González, 2018). ADSCs are involved in regulating the TEM, interact with tumors, and have achieved satisfactory results in the treatment of bladder cancer and breast cancer (Rigotti et al., 2009; Wang et al., 2019). ADSC-derived EVs (ADSC-EVs) have no immunogenicity and have heterogeneous subsets of derived cells, thus obtaining good antitumor properties and representing a good tool for the treatment of tumors. Studies have proven that CD90^low^ADSC-EVs have anticancer activity (Zeng et al., 2019), Li and coauthors loaded miR-16-5p with an anticancer effect into CD90^low^ADSC-EVs and observed that it significantly promoted cancer cell apoptosis and slowed tumor growth (Li et al., 2020). Notably, miR-199a-3p is also an important miRNA involved in tumor regulation that is expressed at high levels in normal liver cells but downregulated in HCC (Callegari et al., 2018). After the inclusion of miR-199a-3p, exosomes secreted by ADSCs (ADSC-exo-199a) effectively targeted mammalian target of rapamycin (mTOR), which increased the sensitivity of tumors to chemotherapeutic drugs and significantly reduced the tumor volume (Lou et al., 2020). ADSC-derived exosome administration provides a new strategy for overcoming chemotherapeutic resistance.

#### 3.2.3. Macrophages

According to many studies, macrophage-derived exosomes (M-EXOs) are indispensable in the communication between cancer cells and macrophages (Liu et al., 2020). As mentioned in the section on macrophage membranes above, macrophages differentiate into M1 and M2 phenotypes with different functions in response to different stimuli (Binenbaum et al., 2018).

Recently, Wang and colleagues compared the differences between M1 exosomes (M1-Exos) and M2 exosomes (M2-Exos). When macrophages were coincubated with these two exosomes, IL-12 and TNF-α levels were increased in the M1-Exos group, while the opposite results were obtained from the M2-Exos group, indicating the proinflammatory effect of M1-Exos. When PTX was loaded into M1-Exos, M1-Exos were used as carriers to deliver chemotherapeutic drugs, and the stimulation of M1-Exos with IFN-γ activated the NF-κB pathway, which created a proinflammatory environment, enhanced the antitumor effect of the drug, and fully exploited the advantages of M1-Exos. The drug-loaded exosomes (PTX-M1-Exos) were more likely to accumulate in the tumor than PTX because M1-Exos replicated the function of the source cells, effectively targeting tumors and activating macrophages to destroy tumors. In addition, the safety of PTX-M1-Exos was so high that it had no discernible organic toxicity, even if double the therapeutic dose was administered (Wang et al., 2019). In another study, an anti-CD47 antibody and anti-SIRP α antibody were modified on M1-Exos. When the drug was administered to the tumor site, it not only blocked CD47 on tumor cells to eliminate the ‘do not eat me’ signal but also targeted SIRP α on macrophages and enhanced the phagocytic activity of macrophages. In addition, M1-Exos also transform M2 macrophages into M1 macrophages, suggesting that M1-Exos have a bright future as an antitumor treatment (Nie et al., 2020).

In addition to the direct drug delivery method described above, M-Exos can also load NPs, improving the properties of NPs and enhancing the delivery of the drug to the desired site. Due to the lack of targets, many treatments for triple-negative breast cancer (TNBC) are ineffective, and the commonly used drug-loaded NPs are also ineffective because of their high toxicity and ability to activate systemic immunity (Yadav et al., 2014; Xu et al., 2018). A new study modified peptides targeting mesenchymal–epithelial transition factor on TNBC on M-Exos and then loaded PLGA NPs (MEP-D) containing the anticancer drug Dox, taking full advantage of the nontoxicity and low immunogenicity of these endogenous vesicles. The exosomes produced by immune cells inherit the tumor targeting ability of their parent cells and efficiently deliver large quantities of drugs to the target tissue. After administration, no tissue damage was observed in sections of the principal organs, and no significant changes were observed in the serum levels of liver-related enzymes, indicating low hepatotoxicity after MEP-D administration (Li et al., 2020).

In recent years, in-depth studies of macrophage-derived EVs have been conducted, which also provides more recent ideas for cancer treatment. M2-Exos were modified to inhibit tumors, and downregulating lncRNA SBF2-AS1 in M2-Exos promoted the expression of miR-122-5p and then inhibited the X-linked inhibitor of apoptosis protein, which ultimately prevented the development of pancreatic cancer (Yin et al., 2020). Therefore, M-Exos have a complex mechanism, and their more effective utilization still requires further research.

#### 3.2.4. Cancer cells

Compared to normal cells, tumor cells contain more lipids, proteins, and nucleic acids, and exosomes secreted by tumor cells also contain these substances and play an important role in the communication between cancer tissues and distant organs. Tumor cell-derived exosomes (TEXs) are similar to their parent cells and interact with their parent cells. Lipids on the tumor cell membrane surface determine the fusion of TEXs with their parent cells (Parolini et al., 2009), and unique proteins on TEXs facilitate their fusion with parent cells (Smyth et al., 2014). When the drug Doxil (a Dox liposome) was loaded into HT1080 exosomes and used to treat HT1080 tumor-bearing mice, the drug-loaded HT1080 exosomes (D-HT1080 exos) displayed strong tumor targeting in vivo. Compared with the group that was directly administered Doxil, the concentration of Dox in the tumor site of the group treated with D-HT1080 exos increased by 2.3 times, and the retention time was also significantly increased (Qiao et al., 2020).

The metastasis of cancer cells not only depends on the characteristics of cells but is also closely related to the microenvironment of the premetastatic niche (PMN), which is conducive to the metastasis of primary tumors. S100A4 advances the formation of PMNs and builds a microenvironment suitable for the survival of malignant tumors, while an S100A4 siRNA (siS100A4) blocks the expression of S100A4 and inhibits tumor growth (Liu et al., 2018; van den Brand et al., 2018). Using the advantages of bionics, Zhao et al. conjugated cationic bovine serum albumin and siS100A4 (CBSA/siS100A4) and then encapsulated them within autologous breast cancer-derived exosomes (OCC@EXOs) to form a novel biomimetic carrier (CBSA/siS100A4@Exosomes). Due to the lung targeting properties of integrins on the OCC@EXO membrane, CBSA/siS100A4@Exosomes had a stronger ability to target lung tissue than liposome-coated CBSA/siS100A4, increasing drug release and adhesion to lung PMNs (Wortzel et al., 2019). In addition, OCC@EXOs reduced the cytotoxicity and immunogenicity of DDSs to a very low level and exhibited strong affinity for the parent cancer cells, which increased the release of drugs at the target and enhanced the anticancer effect (Zhao et al., 2020).

Cancer immunotherapy is an emerging treatment method. In contrast to chemotherapy and RT, cancer immunotherapy works by enhancing the ability of the immune system to fight against tumors, with minimal side effects. Proteins on TEXs, such as CD63, CD81, liposome-associated membrane protein 1 (LAMP1), MHC-I, MHC-II, and Annexin II, interact with ligands on DCs to promote the binding of DCs to TEXs (Hong et al., 2014; Gu et al., 2015). Under ideal conditions, the combination of an immune adjuvant with TEXs improves the immunogenicity of DCs and the efficacy of immunotherapy. High mobility group nucleosome-binding protein 1 (HMGN1) is a potent immune adjuvant that activates DCs and induces a sustained immune response (Yang et al., 2012). In a recent study, TEXs (TEX-N1ND) were modified with the functional N-terminus of HMGN1 (N1ND) to deliver TAAs and N1ND to DCs together, thereby activating DCs, promoting DC migration to lymph nodes, and increasing the generation of memory TLs. This treatment substantially increased the strength of antitumor immunity, remodeled the TME of orthotopic HCC in mice with a deficiency in original immunogenicity, and delayed tumor growth (Zuo et al., 2020). In addition, some miRNAs, such as miR-142 and let-7i, also activate DCs and TLs, and tumor cell-derived EVs loaded with multiple miRNAs have been used to deliver tumor-specific antigens to DCs, significantly affecting the maturation of DCs and the activity of CTLs, reducing tumor volume and prolonging the survival of mice (Khani et al., 2021). This attempt to activate DCs using TEXs transporting antigens and exogenous substances has promoted the development of DC immunotherapy.

#### 3.2.5. Other sources

In addition to EVs produced by these cells (Table 2), several other sources of EVs have been attempted to be used for targeted cancer therapy in recent years, such as HEK293 cell-derived exosomes (HEK293-Exos), milk-derived exosomes secreted by mammary gland epithelial cells, and OMVs. HEK293 cells were transfected with miR-204-5p, which inhibits tumor growth and metastasis, and secreted exosomes stably expressing miR-204-5p. Epidermal growth factor and GE11 peptides on HEK293-Exos recognize epidermal growth factor ligand on tumor cells, enabling the carrier to target tumors, and then miR-204-5p effectively inhibited tumor growth in mice, increased apoptosis induced by 5-fluorouracil, and reversed drug resistance to chemotherapy (Yao et al., 2020).

**Table 2.** Extracellular vesicles as delivery carriers for targeted cancer therapy.

Donor	Cargo	Cancer type	Target	Outcome	Ref
DCs	Ovalbumin, LPS and IFN-γ	T cell carcinoma	Macrophages, DCs and T cells	Boosted both innate and adaptive immunity	(Matsumoto et al., 2020)
Breast cancer cells	miR-126	Lung cancer	A549 cells	Inhibiting lung metastasis	(Nie et al., 2020)
BM-MSCs	miR-375	Cervical cancer	Cervical cancer cells	Discover new biomarkers for cervical cancer treatment	(Ding et al., 2020)
Human liver stem cells	miR-145 and miR-200	Renal cell carcinoma	Renal cancer stem cells	Inhibiting tumor growth	(Brossa et al., 2020)
HEK-293 cells	HN3 protein	Liver cancer	GPC3 + HuH-7 cancer cells	Effectively targeting liver cancer cells and inhibiting tumor growth	(He et al., 2020)
DCs	CD9 and CD63	Lung cancer	T cells and T cell subset populations	Induced immune responses	(Than et al., 2020)
DCs	E749-57 peptide	Cervical cancer	CD8+ T cells	Induced protective immunity responses to cervical cancer	(Chen et al., 2018)
Human breast cancer cells	PTX-linoleic acid prodrug and CuB	Breast cancer	CTCs	Inhibiting tumor regression and metastasis	(Wang et al., 2020)
BM-MSCs	Let-7	Lung cancer	KDM3A/DCLK1/FXYD3 axis	Significantly suppressing cancer proliferation, migration and invasion	(Liu et al., 2021)
Breast cancer cells	miRNAs (Let-7i, miR-142 and, miR-155)	Breast cancer	DCs and T cells	Inhibiting of solid tumors	(Khani et al., 2021)
Hepatocellula carcinoma cells	miR30a-3p	Hepatocellular carcinoma	SNAP23 gene	Effectively attenuating HCC migration, invasion, and metastasis	(Liu et al., 2021)

Exosomes derived from bovine milk have the advantages of a low cost, high yield, easy extraction, and good biological and physical stability and are good tools for oncology drug delivery (Admyre et al., 2007; Chen et al., 2020). However, the lack of targeting is also an issue that must be addressed (Melnik et al., 2014). Bovine milk exosomes were modified with hyaluronic acid (HA), which targets CD44 overexpressed on tumors, and then loaded with Dox to form a new carrier (HA-mExo-Dox) that specifically targets CD44-positive tumor cells and allow tumor cells to efficiently take up Dox (Li et al., 2020).

OMVs facilitate communication between bacteria and the environment and are biodegradable and targeted. OMVs, as attractive carriers, carry immunostimulatory factors and induce appropriate immune responses, showing great potential in tumor immunotherapy. Dox-carrying OMVs (Dox-OMVs) from attenuated *Klebsiella pneumoniae* not only interact with epithelial cells to initiate immune signals and recruit immune cells but also directly interact with macrophages to activate the immune system, induce antitumor immunity and increase the toxicity toward non-small cell lung cancer (Kuerban et al., 2020).

## 4. Future challenges

Many advantages of cell membrane-coated nanoparticles and EVs have been reported, especially in terms of targeting and biocompatibility. Current synthetic DDSs are essentially foreign materials with potential toxicity and immunogenicity, while cell membranes and EVs are endogenous and are deemed to be biocompatible and have multiple biological functions that are similar to the source cell. However, some issues remain to be addressed for these carriers to continue to evolve and make the transition from the laboratory to the clinic.

First, the question of yield must be addressed. Existing separation technologies not only produce a small amount of EVS but are also expensive for large-scale production. Therefore, more advanced large-scale production methods are needed to continue to expand the application of EVs. Initially, scientists increased the release of vesicles by adding exogenous compounds to the cells from which EVs are derived (Allan et al., 1980). In recent years, an increasing number of studies on EV mimetics, which are artificial vesicles obtained from the membrane broken by extrusion, have been conducted to solve the yield problem (Sil et al., 2020). The properties of EV mimetics are similar to those of natural EVs, with better scalability and higher bioavailability. The same number of THP-1 cells produces 2.5 times more simulated exosomes than natural exosomes, and the former has higher encapsulation and drug release rates (Pisano et al., 2020). The process used to prepare cell membranes is mature and results in a much higher yield than the preparation of exosomes, but the separation and purification schemes also must be adjusted and optimized because a large number of cells still need to be cultured to obtain a sufficient number of membranes, and the preparation process still needs to be simplified (Li et al., 2018).

The processes of modification and loading may alter the original properties of the cell membrane. For RBCMs that lack a targeting capability, the membranes must be endowed with the ability to reach the target site for the release of therapeutic cargoes, but the modification of the membrane is likely to change its original structure and reduce the biocompatibility of the carrier. PLTM is highly sensitive, and a suitable loading scheme must be identified to ensure sufficient drug loading and safe delivery of the drug to the target site (Wang et al., 2020). The stability and toxicity of modified or drug-loaded EVs also require further exploration, especially as carriers for cancer nanomedicines. The appropriate drug loading method should be selected to effectively load the drug into the EVs with the minimum ratio of carrier to drug to achieve the desired dose and release profile (Susa et al., 2019).

In addition, the complex mechanism of the transport of cell membranes from different sources as carriers in vivo is not completely understood and requires further study (Li et al., 2020). For example, the delivery of therapeutic molecules by carriers based on white cell membranes may activate components of the immune system and trigger inflammation (Jin et al., 2018). When CCM is used, it may induce cancer development in the body if the genetic material from the parent cancer cells is not completely eliminated. Methods for the purification and characterization of EVs vary from laboratory to laboratory, and different methods may result in confusion regarding the subgroups and physicochemical properties of EVs. Therefore, researchers can share data and reasonably develop a unified standardized procedure with excellent repeatability for the quality control of EVs.

**Figure 8.**
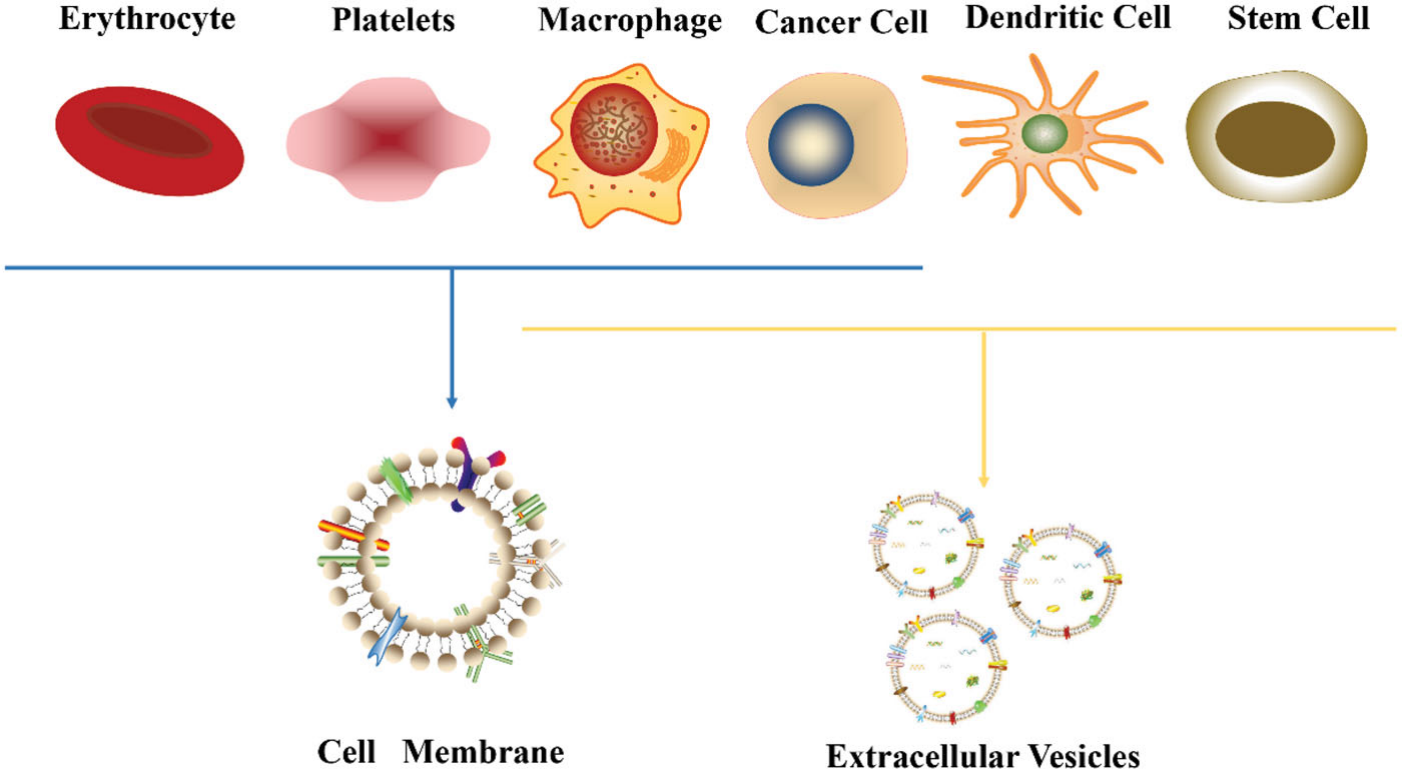
Common cell types of carriers for tumor targeted therapy.

Both the cell membrane and EVs enable carriers to effectively cross biological barriers and target cancer tissues. Some cells can not only be used to extract membranes to prepare carriers but also for the isolation of their EVs to transport drugs (Figure 8). The extraction and preparation of the cell membrane is relatively easy, but the targeting ability may be impaired due to the loss of proteins during membrane extraction. Although the preparation of EVs is challenging, they generally retain the complete membrane components, and thus they have excellent targeting ability (Xia et al., 2020). Therefore, the appropriate carrier must be selected according to the purpose of the experiment to improve the therapeutic effect as much as possible. Differences between cell membrane vesicles and EVs are summarized in Table 3.

**Table 3.** Comparison of cell membrane vesicles and extracellular vesicles.

	Cell membrane vesicles	Extracellular vesicles
Source	Extrusion of cell membrane	Secretion by cells
Size (diameter)	100–400 nm	20 nm–2 μm
Separation	Separation process of membrane and intramembrane mixture	Separation of extracellular vesicles and source cells
Cargo loading	Nanoparticles or direct loading of drugs	Drugs are usually loaded directly.
Source of cells	Red blood cell, white blood cell, platelet, cancer cell, fibroblast, bacterial, etc.	Dendritic cell, stem cell, macrophage, cancer cell, HEK293 cell, bacterial outer membrane vesicles, etc.
Advantages	Long-term circulation; Good biological barrier permeability; Efficient cell fusion; Large production.	Low immunogenicity, non-cytotoxicity, high biocompatibility; Intrinsic tumor targeting; Efficient cellular uptake.

## 5. Conclusions

Although cell-based DDSs face many challenges, their powerful advantage of ‘mimicking nature’ still overcomes many of the disadvantages of traditional DDSs and provides a more effective strategy for cancer treatment. The substance on the surface of the new DDS takes advantage of the natural characteristics of cells, such as the enrichment of targeted proteins, long-term circulation in the body, ability to pass through biological barriers, interactions with other cells, and reduced tissue and cell toxicity, which effectively protect the cargo carried and substantially improve the therapeutic effect. With the rapid development of pharmacology, material science, bioinformatics, proteomics and nanotechnology, the combination of DDSs and cells is expected to overcome many obstacles, change the current medical technology, and provide new horizons for targeted cancer therapy.
